# Exploring the Potentials of *Lysinibacillus sphaericus* ZA9 for Plant Growth Promotion and Biocontrol Activities against Phytopathogenic Fungi

**DOI:** 10.3389/fmicb.2017.01477

**Published:** 2017-08-17

**Authors:** Zakira Naureen, Najeeb Ur Rehman, Hidayat Hussain, Javid Hussain, Syed A. Gilani, Saif K. Al Housni, Fazal Mabood, Abdul L. Khan, Saima Farooq, Ghulam Abbas, Ahmed A. Harrasi

**Affiliations:** ^1^Department of Biological Sciences and Chemistry, College of Arts and Sciences, University of Nizwa Nizwa, Oman; ^2^UoN Chair of Oman’s Medicinal Plants and Marine Natural Products, University of Nizwa Nizwa, Oman

**Keywords:** *Lysinibacillus*, antifungal metabolites, 2-pentyl-4-quinolinecarboxylic acid, IAA, HCN, biocontrol

## Abstract

There is an ongoing hunt for biologically active compounds that can combat phytopathogenic fungi and improve plant growth without causing any hazards to the environment. Consequently the present study aims at deciphering the plant growth promotion and antifungal capability of *Lysinibacillus sphaericus* ZA9. The bacterium was previously isolated and identified in our laboratory from maize rhizosphere using 16S rRNA gene sequencing. The test bacterium *L. sphaericus* ZA9 was found to produce high quantity of IAA (697 μg/ mL); siderophores (195.79 μg/ mL), HCN and hydrolytic enzyme as compared to the reference strain *Bacillus sphaericus* Z2-7. The bacterium was also capable of solubilizing silicates (Si), phosphates (P), and potassium (K). The bacterium enhanced the seedling vigor and germination of seeds pretreated with it and promoted the shoot length of both cucumber and tomato seeds in greenhouse experiment. *L. sphaericus* ZA9 and its cell free culture supernatant showed varied antagonistic behavior against *Alternaria alternata, Curvularia lunata, Aspergillus* sp., *Sclerotinia* sp., *Bipolaris spicifera, Trichophyton* sp. Fermentation broth culture of *L. sphaericus* ZA9 was then used to isolate antifungal metabolites by silica column chromatography. Identification and determination of antifungal compounds was carried out by Thin-layer chromatography (TLC) followed by NMR spectroscopy. Two compounds were isolated and identified as 2-pentyl-4-quinolinecarboxylic acid (C_15_H_17_NO_2_) which is a quinoline alkaloid and 1- methylcyclohexene which is a cycloalkene. Compound 1; 2-Penthyl-4-quinolinecarboxylic acid was found to be highly antagonistic against most of the fungi tested as compared to the bacterium itself. Its activity was comparable to that of fungicide Benlate, while compound 2; 1- methylcyclohexene did not show any antifungal activity.

## Introduction

Phytopathogens are a continuous challenge for the farming population of the world besides scarcity of water and deteriorating soil fertility levels. Presence or absence of phytopathogens directly affects plant health and the resulting productivity. Almost all the crops are affected by one or more pathogens and the farmers have no other option but to spray pesticides which are not only accumulating in our plant resources but also in our water bodies and aquatic life, a phenomenon known as biomagnification (Goutte et al., 2015). For this reason, there is an ongoing hunt of natural compounds produced by biological organisms which may alleviate the need of chemical fungicide and pesticides for controlling plant pathogens and improving crop productivity (Naureen et al., 2015). Several bacterial and fungal species have been reported by many research groups which cannot only improve plant growth and productivity but are excellent in combating plant pathogens in laboratory and greenhouse conditions (Gupta et al., 2001; Naureen et al., 2009, 2015; Hassan et al., 2010). However, the performance of many of these plant growth promoting biocontrol (PGPB) agents is highly compromised when they are applied in fields under natural environmental conditions (Ojiambo and Scherm, 2006). For this reason, in addition to exploring the potentials of PGPB there is a shift in research to isolation and application of natural compounds from such biocontrol agents which cannot only combat phytopathogens but also are unaffected by varying environmental conditions in the field (Dayan et al., 2009). For instance, Blasticidan S; Streptomycin; Validamycin; Natamycin etc. isolated from *Streptomyces* sp. are used as foliar sprays for eradicating rice blast fungus *Magnaporthe grisea*; *Rhizoctonia solani*; Powdery mildew and a wide range of other phytopathogenic fungi (Dayan et al., 2009; Stokowa-Soltys and Jeżowska-Bojczuk, 2013). Most of the natural antifungal compounds used as foliar sprays have been isolated from *Streptomyces* sp., *Pseudomonas* sp., *Bacillus* sp., and *Brevibacillus* sp. etc. and have proven to be useful against notorious phytopathogenic fungi (Kadioglu et al., 2011; Lang and Buchbauer, 2012).

*Lysinibacillus sphaericus* ZA9 is one of the important bacterial species which has been known to produce antimalarial and larvicidal compounds such as Cry48/Cry49 (White and Lotay, 1980; Claus and Berkeley, 1986; Ahmed et al., 2007; Jones et al., 2007) and the S-layer protein (Lozano et al., 2011; Lozano and Dussán, 2013) which kill the mosquito larvae. It is a well-known Bio-insecticide and is used as part of vector control programs against malaria, filariasis, yellow fever, dengue fever, and West Nile virus (Berry, 2012). However, there are scarce reports for the involvement of this bacterium in plant growth promotion and production of antifungal metabolites. Consequently, the present study aims to evaluate maize root associated *L. sphaericus* strain for production of secondary metabolites that can promote plant growth and antagonize selected phytopathogenic fungi such as *Alternaria alternata, Curvularia lunata, Aspergillus* sp., *Sclerotinia* sp., *Bipolaris spicifera, Trichophyton* sp.

## Materials and Methods

### Bacterial Growth and Culture Conditions

The bacterium *L. sphaericus* ZA9 was isolated previously and identified by 16S rRNA gene sequencing and analysis in our laboratory from maize rhizosphere (Accession number KT335955). The reference strain *Bacillus cereus* Z2-7 (Yasmin et al., 2004) was obtained from BIRCEN culture collection National Institute for Biotechnology and Genetic Engineering (NIBGE), Faisalabad, Pakistan. The bacterial strains were stored in glycerol stocks at 4°C. The stored cultures were obtained and refreshed in 50 mL of Nutrient Broth (NB). The broth cultures were kept in an incubator shaker at 28±2°C for 1–2 days. The fresh bacterial cultures were used for further studies.

### Plant Growth Promoting Activities of *Lysinibacillus sphaericus* ZA9

#### Indole Acetic Acid (IAA) Production

Salkowski reagent (Patten and Glick, 2002) was used for IAA detection and quantification. *L. sphaericus* ZA9 and *B. cereus* Z2-7 were grown separately in 100 mL NB with and without 0.5 g l^-1^ tryptophan (precursor of IAA) in darkness (Mergeay et al., 1985) for 3 days at 28±2°C in an incubator shaker at 120 rpm. Bacterial culture suspensions were centrifuged (30 min at 3220 × *g*) and 0.2 mL of the supernatant was mixed with 1 mL Salkowski’s reagent (50 mL of 35% HClO4, 1 mL 0.5 M FeCl3). Development of pink color after 30 min indicated IAA production. The absorbance of pink color was read at 530 nm using a ELISA reader. The IAA concentration was determined using a calibration curve of pure IAA as a standard following the linear regression analysis.

#### Silicate, Phosphate, and Potassium Solubilization

For silicate solubilization assay pure bacterial cultures of *L. sphaericus* ZA9 and *B. cereus* Z2-7 were streaked on agar plates containing silicate medium [Peptone 1 g/L; Yeast extract 1 g/L; Glucose 20 g/L, (NH_4_)_2_SO_4_ 0.05 g/L; MgCl_2_; Magnesium trisilicate 25g/ L; Bacteriological agar 20 g/L; pH 6.6] (Bunt and Rovira, 1955; Naureen et al., 2015). For phosphate and potassium solubilization the pure bacterial cultures were streaked on agar plates containing Pikovskaya’s medium (pH 8.44) amended with bromocresol purple (0.1 g/L) and Alexandrove’s medium (pH 7.4), respectively (Pikovskaya, 1948; Aleksandrov et al., 1967; Hassan et al., 2010; Lü and Huang, 2010; Naureen et al., 2015). Plates for each solubilization assay were incubated for 4–7 days at 28 ± 2°C. Average zone diameters for both the test and reference strains were calculated for each experiment run in triplicates.

#### Organic Acid Production

The production of organic acid by the *L. sphaericus* ZA9 and *B. cereus* Z2-7 was detected on Sucrose Tryptone medium as described by Cunningham and Kuiack (1992) with a few modifications. Phenol red was an indicator instead of Alizarin red.

#### Effect of *Lysinibacillus sphaericus* ZA9 on Germination and Growth of Tomato and Cucumber Plants

##### Preparation of bacterial inoculum for seed treatment

Bacterial strains *L. sphaericus* ZA9 and *B. cereus* Z2-7 were cultured in 250 mL conical flasks containing 200 mL Nutrient broth medium on an incubator shaker at 120 rpm for 2 days. Bacterial cells were then collected by centrifugation of at 13000 rpm for 10 min at 4°C. The bacterial pellets were resuspended in autoclaved distilled water and the number of cells adjusted to 10^9^ cells/mL.

##### Seed treatment

Tomato seeds of the variety Majestic F1 and cucumber seeds of the variety Beth alpha were procured from local market. Approximately 100 seeds for each plant were surface sterilized by immersing in 5% NaOCl for 1 min followed by washing with autoclaved distilled water for 5 min. Seeds were then spread on autoclaved filter paper and 50 dried seeds were then soaked in each bacterial cell suspension for 30 min. The treated seeds were then spread on sterile petri dishes and dried overnight in a clean bench.

##### Seed germination and seedling vigor assay

The treated dried seeds of tomato and cucumber as prepared above were then allowed to germinate on sterile moist filter paper beds in sterile petri dishes. Fifty seeds of tomato and cucumber inoculated with each bacterial strain were germinated in 9 cm petri dishes (10 seeds/petri dish). Seeds treated with autoclaved distilled water served as control. The petri dishes were then sealed with parafilm and kept in dark for 5–7 days at 28±2°C. The filter paper beds were kept moistened by spraying autoclaved distilled water on alternate days (Islam et al., 2016).

Seed germination was considered when the radicles were half of the seed length. The experiment design was completely randomized with five replicates for each bacterium for each of the plant and each replicate containing 10 seeds. The germination percentage was recorded after 5 days as follows.

$$\begin{array}{c} \mathrm{Germination}\mathrm{percentage}\;=(\mathrm{numbers}\mathrm{of}\mathrm{seed}germinated/total\mathrm{number}\mathrm{of}\mathrm{seeds})\;\times \;100.\\ \mathrm{Vigor}\mathrm{Index}\;=\;\%\mathrm{germination}\times \mathrm{total}\mathrm{plant}\mathrm{length}.\; \end{array}$$

##### Greenhouse experiment

In order to evaluate the effect of *L. sphaericus* ZA9 on growth of tomato and cucumber plants; seed inoculation and germination was carried out as mentioned above. Three pre-germinated seeds of tomato and cucumber were then sown in sterilized pots containing 200 g of autoclaved soil. Ten pots/bacterium/plant type were placed in a completely randomized design. The germinated seeds from the above experiments were then used for greenhouse experiment. The pots were kept in a greenhouse at optimum conditions for 1 month. The root and shoot length of the seedling were recorded 10 and 21 days post plantation (dpp). Plants from five pots were harvested at 10 and 21 dpp, respectively. The uninoculated surface sterilized seeds of tomato and cucumber grown in same conditions served as control.

### Antagonistic Activity of *Lysinibacillus sphaericus* ZA9

#### Dual Culture Assay

Antagonistic activity of *L. sphaericus* ZA9 and the reference strain *B. cereus* Z2-7 was checked against selected phytopathogenic fungi (*A. alternata, C. lunata, Aspergillus* sp., *Sclerotinia* sp., *B. spicifera, Trichophyton* sp.) using dual culture assays as described by Naureen et al. (2009) and Hassan et al. (2010). Percentage mycelial growth Inhibition was calculated by the formula [1- fungus growth diameter in test/fungus growth diameter in control] × 100. The measurements were taken after 7 days. The experiment was run in triplicates.

#### Antagonistic Activity of Bacterial Culture Supernatant

To detect the antagonistic activity of bacterial culture supernatant 1 mL of fresh bacterial cultures of *L. sphaericus* ZA9 and *B. cereus* Z2-7 were centrifuged at 13000 rpm at 4°C for 10 min. The resulting supernatant was passed through a 0.2 μm filter. Sterile filter paper disks were dipped in the supernatant and kept at two places on PDA plates containing 10 mm disk of actively growing mycelia of respective fungal cultures placed in the center. Filter paper disks dipped in autoclaved distilled water and 1000 ppm fungicide Benlate served as control.

#### Production of Secondary Metabolites by *Lysinibacillus sphaericus* ZA9

Pure cultures of *L. sphaericus* ZA9 and *B. cereus* Z2-7 were checked for production of secondary metabolites such as siderophores (Schwyn and Neilands, 1987; Sharma and Johri, 2002), HCN (Lorck, 1948), hydrolytic enzymes such as chitinases, proteases, lipases, and cellulases (Naureen et al., 2009, 2015) as per standard protocols. Quantification of secondary metabolites produced by *L. sphaericus* ZA9 and *B. cereus* Z2-7 *was* carried out according to Naureen et al., 2009.

### Extraction of Antifungal Substances

Fresh culture of *L. sphaericus* ZA9 was prepared in nutrient broth. 50 mL of broth cultures was centrifuged at 13000 rpm for 5 min at 4°C (Gupta et al., 2001). The supernatant was then transferred into a new 50 mL tube to purify antifungal compounds (Strom et al., 2002; Schwenninger et al., 2008).

#### Extraction and Isolation

The culture supernatant was then extracted with ethyl acetate to afford 0.6 g of a residue that was separated into five fractions (LSF_1_–LSF_5_) by column chromatography (CC) on silica gel, using gradients of *n*-hexane, *n*-hexane/EtOAc, EtOAc/MeOH and finally, pure MeOH as mobile phases. LSF_4_ was further subject to column chromatography by silica gel with EtoAc/MeOH (9:1 and 8:2) as eluents to give one new compound **1** (2.6 mg) and one known compound **2**.

**2-penthyl-4-quinolinecarboxylic acid:** Cream crystalline powder; mp 195.2°C; IR (MeOH): (cm^-1^) 3410 (OH), 1715 (C = O), 1580 and 1420 (benzene ring); ^1^H NMR (600 MHz, CD_3_OD): δ = ppm 0.85 (*t*, 3H, *J* = 7.0 Hz), 1.32–134 (*m*, 4H), 1.57 (*m*, 2H), 2.61 (*t*, 2H, *J* = 7.8 Hz), 8.33 (dd, 1H, *J* = 7.8, 0.6 Hz, H-5), 7.57 (*t*, 1H, *J* = 7.8 Hz, H-7), 7.32 (*t*, 1H, *J* = 7.8 Hz, H-6), 7.38 (*d*, 1H, *J* = 8.4 Hz, H-8), 6.18 (*s*, 1H, H-3), 11.98 (*s*, 1H); ^13^C NMR (125 MHz, CD_3_OD): δ = 14.3 (C-5′, CH_3_), 28.5 (C-4′, CH_2_), 29.6 (C-3′, CH_2_), 31.6 (C-2′, CH_2_), 34.4 (C-1′, CH_2_), 108.8 (C-3, CH), 117.2 (C-8, CH), 123.6 (C-6, CH), 124.9 (C-4, C), 125.9 (C-5, CH), 131.9 (C-7, CH), 139.7 (C-4a, C), 153.2 (C-8a, CH), 165.1 (C-2, C), 178.8 (C = O). ESIMS: *m/z* (*rel. int*.): *m/z* 266.12 [M + Na].

### Antifungal Activity of 2-Penthyl-4-Quinolinecarboxylic Acid and 1 Methyl Cyclohexene

To check the antifungal activity of the isolated compound against the phytopathogens a modified dual culture assay was used.

Briefly, 10 mm disks of pure culture of pathogenic fungi grown on potato dextrose agar (PDA) were placed at the center of a Petri dish containing the appropriate test medium (including PDA and Nutrient Agar). A circular inoculum, made with a 6 cm diameter Petri dish dipped in a 1% suspension of Compound 1, 2-penthyl-4-quinolinecarboxylic acid and Compound 2; 1 methyl cyclohexene were placed surrounding the fungal culture. Autoclaved distilled water was used as control. Plates were incubated for 7 days at 30 ± 1°C and inhibition of pathogen growth calculated by using the following formula.

$$\begin{array}{c} \%\mathrm{Inhibition}\;=\;[1-(\mathrm{Fungal}growth/control\mathrm{growth})]\;\times \;100 \end{array}$$

There were three replicates per treatment and each experiment was repeated three time.

Alternatively, mycelial disks of 10 mm were placed on PDA agar plates. Autoclaved filter paper disks immersed in a solution of 1% 2-penthyl-4-quinolinecarboxylic acid and 1 methyl cyclohexene were placed at 2 points in each plate separately. Disks dipped in autoclaved distilled water and 1000 ppm solution of fungicide Benlate served as control. The plates were incubated at 30 ± 1°C for 7 days. Zones of inhibition were recorded as mentioned above.

### Statistical Analysis

Statistical analyses were performed using SYSTAT version 13.1 and Microsoft Office Excel 2010. A completely randomized design was used in all plant related experiments. At least three replicates were used for all experiments related to *in vitro* evaluation of *L. sphaericus* ZA9 for plant growth promotion and biocontrol activities while 10 replicates were used for greenhouse experiment and each replicate contained three seedlings. Each experiment was repeated three times and the data presented is average of all. Treatments were compared via ANOVA using the least significant difference test (LSD) at 5% (*p* ≤ 0.05) probability level. The differences in germination percentage and seedling vigor studies were calculated using ANOVA and Tukeys HSD *post hoc* test at a confidence level of 97%.

## Results

*Lysinibacillus sphaericus* bacterium previously isolated and identified in our lab using 16S rRNA gene sequencing from maize rhizosphere was screened for its abilities to promote plant growth and control phytopathogenic fungi as compared to the reference strain *B. cereus* Z2-7 that has been previously reported by us as a PGPB strain.

### Plant Growth Promoting Activities of *Lysinibacillus sphaericus* ZA9

As shown in **Table 1** the *L. sphaericus* ZA9 was found to produce high quantities of IAA as compared to the reference strain. It was also observed to solubilize silicates, phosphates and potassium and produce bigger clearing zones of Phosphate solubilization as compared to the reference strain *B. cereus* Z2-7. Both of the test and reference strains were capable of producing organic acids as shown in **Table 1A**.

**Table 1A:** Plant growth promoting activities of *Lysinibacillus sphaericus* ZA9.

Bacterial isolate	IAA μg/mL	Zone diameter in cm			Acid^∗^ production
		Silicate solubilization	Phosphate solubilization	Potash solubilization	
*Lysinibacillus sphaericus* ZA 9	697 ± 10.2	4.1 ± 0.32	5.0 ± 0.4	5.0 ± 0.8	**+++**
*Bacillus cereus Z2-7*	284 ± 4.8	4.0 ± 0.62	1.2 ± 0.25	4.2 ± 0.68	**+++**

***^∗^**Conversion of Phenol red to yellow.*

*A complete yellow color is indicated by +++.*

In order to evaluate the effect of *L. sphaericus* ZA9 on tomato and cucumber, seeds were pretreated with the test bacterium and reference strain, respectively, and the seed germination, seedling length and vigor index were compared with that of control (**Table 1B**).

**Table 1B:** Effect of *L. sphaericus ZA9* on germination percentage and seedling vigor of tomato and cucumber plants.

Plants type	Treatments	Germination % ^∗^*p* = 0.000	Average seedling length mm ^∗^*p* = 0.000	Vigor index ^∗^*P* = 0.0003
Tomato	Uninoculated control	85 ± 2.31a	23.2 ± 1.2a	1972a
	*Bacillus cereus* Z2-7	92 ± 3.46ab	44.6 ± 1.41b	4103.2b
	*Lysinibacillus sphaericus* ZA 9	95 ± 1.42b	52.9 ± 3.2c	5025.5c
Cucumber	Uninoculated control	82 ± 1.64a	62.6 ± 0.86a	5133a
	*Bacillus cereus* Z2-7	89 ± 2.44b	76.4 ± 1.34b	6799.6b
	*Lysinibacillus sphaericus* ZA 9	91 ± 1.26b	83.2 ± 2.26c	7571.2b

*^∗^ANOVA.*

*The letters a, b, c, represent significant difference between the groups as calculated by Tukey’s HSD test.*

Eighty five percent of uninoculated tomato seeds; while 95% of the tomato seeds inoculated with *L. sphaericus* ZA9 and 92% seeds inoculated with *B. cereus* Z2-7 were observed to germinate. This means both of the test and reference bacteria enhanced the seed germination by 10 and 7%, respectively, as compared to uninoculated control seeds. Statistical analysis revealed that *L. sphaericus* ZA9 had a highly significant effect (*p* ≤ 0.05) on seed germination of tomato as compared to uninoculated seeds. However, there was no significant difference between *L. sphaericus* ZA9 and *B. cereus* Z2-7 (*p* ≥ 0.05) with respect to enhancement of seed germination (**Table 1B**). Average seedling length and vigor index of tomato seedlings treated with *L. sphaericus* ZA9 and *B. cereus* Z2-7 was significantly higher (*p* ≤ 0.05) than uninoculated control.

In case of cucumber plants *L. sphaericus* ZA9 significantly enhanced germination percentage; average seedling length and vigor index as compared to the uninoculated control seeds (*p* ≤ 0.05). However, there were no significant differences between the effect of *L. sphaericus* ZA9 and *B. cereus* Z2-7 (*p* ≥ 0.05) (**Table 1A**).

**FIGURE 1:**
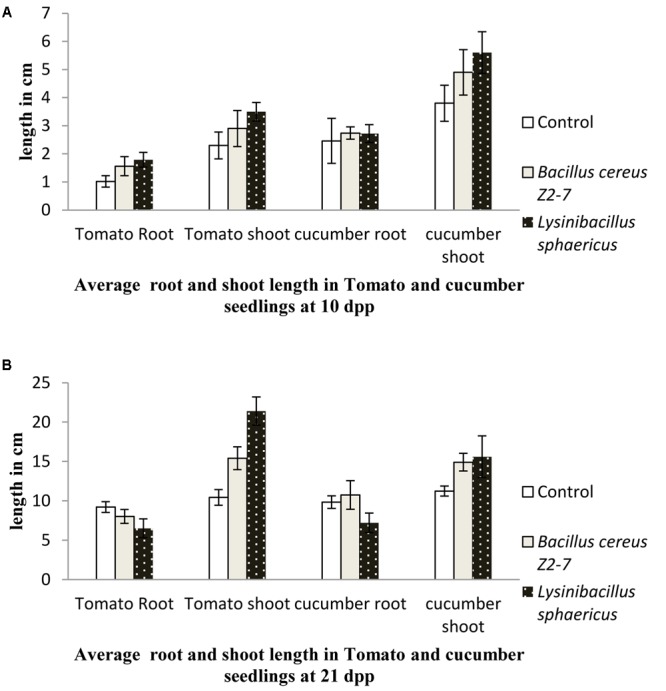
Effect of *Lysinibacillus sphaericus* on growth of *Lycopersicon esculentum* (Tomato) and *Cucumis sativus* (Cucumber) seedlings after 10 days **(A)** and 21 days **(B)** as compared to uninoculated control and reference strain *Bacillus cereus*.

For plants grown in greenhouse conditions no significant differences in root and shoot length of tomato seedlings treated with *L. sphaericus* ZA9 and *B. cereus* Z2-7 were observed as compared to uninoculated control 10 days post plantation (*p* ≥ 0.05). However, *L. sphaericus* ZA9 was observed to significantly enhance the shoot length of tomato seedlings 21 dpp as compared to uninoculated seedlings and those treated with *B. cereus* Z2-9 (*p* ≤ 0.05) (**Figures 1A,B**).

There were no significant differences in cucumber root length for seedlings treated with test and reference bacteria as compared to control 10 dpp (*p* ≥ 0.05), however, at later stages, i.e., 21 dpp root length of cucumber seedlings treated with *L. sphaericus* ZA9 was significantly lower than that of the reference strain and control (*p* ≤ 0.05). While shoot length of cucumber seedlings treated with *L. sphaericus* ZA9 was significantly higher than that of uninoculated control 10 and 21 days post plantation (*p* ≤ 0.05) (**Figures 1A,B**).

### Antagonistic Activity of *Lysinibacillus sphaericus* ZA9

As shown in **Table 2** the test bacterium *L. sphaericus* ZA9 showed higher inhibition of mycelial growth of *A. alternata, Sclerotinia* sp., *Aspergillus* sp., and *Trichophyton* sp. as compared to the reference strain *B. cereus* Z2-7. While the *B. cereus* Z2-7 showed greater mycelial inhibition of *C. lunata* and *B. spicifera*.

**Table 2:** Antagonistic activity of *L. sphaericus* ZA9 and *Bacillus cereus* Z2-7 against different fungi as detected by dual culture assays.

Fungal strains	Percentage Mycelia Inhibition by			
	*Lysinibacillus sphaericus* ZA9	*Lysinibacillus sphaericus* culture supernatant	*Bacillus cereus* Z2-7	*Bacillus cereus* culture supernatant
*Alternaria alternata*	100 ± 0.0	100 ± 0.0	89 ± 3.42	92 ± 3.0
*Curvularia lunata*	29.41 ± 2.32	27.98 ± 2.20	47.23 ± 2.84	46.54 ± 4.0
*Sclerotinia* sp.	69.41 ± 3.29	70 ± 1.61	38.37 ± 1.52	40.22 ± 1.06
*Aspergillus* sp.	53.84 ± 1.18	52.28 ± 2.62	42.5 ± 0.87	46.34 ± 2.54
*Bipolaris spicifera*	13.79 ± 2.13	9.87 ± 1.0	26.21 ± 0.45	25.00 ± 1.44
*Trichophyton* sp.	100 ± 0.0	97.23 ± 3.64	59.32 ± 3.76	58.0 ± 2.65

Cell free culture supernatants (CFCS) of *L. sphaericus* ZA9 and *B. cereus* Z2-7 showed similar antagonistic activities as that of whole bacteria cells with a few exceptions. For instance the CFCS of *B. cereus* Z2-7 exhibited slightly higher antagonistic activity against *A. alternata, Sclerotinia* sp., and *Aspergillus* sp. as compared to that of the whole bacterial cell. On the other hand the CFCS of *L. sphaericus* ZA9 exhibited almost equal or slightly lower inhibition than that of the whole bacterium for all fungi tested.

**FIGURE 2:**
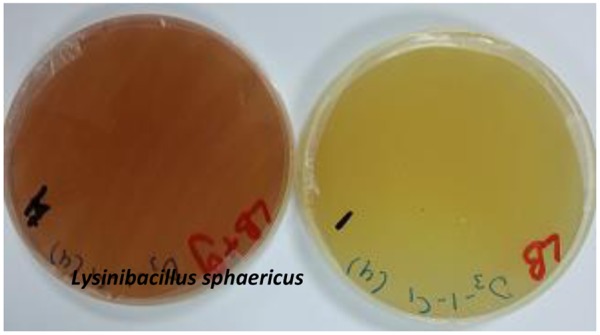
HCN production by *L. sphaericus* ZA9 on Luria Bertani agar medium amended with glycine.

**Table 3** shows the data regarding production of secondary metabolites and hydrolytic enzymes. The *L. sphaericus* ZA9 was found to produce more siderophores, HCN (**Figure 2**) and hydrolytic enzymes as compared to *B. cereus* ZA9.

**Table 3:** Production of secondary metabolites and hydrolytic enzymes by *Lysinibacillus sphaericus*.

Bacterial isolate	Siderophore μg/mL	HCN	Zone diameter in cm			
			Chitinases	Protease	Lipase	Cellulases
*Lysinibacillus sphaericus* ZA 9 (Accession number KT335955)	195.79 ± 8.4	++++	6.1 ± 2.5	7.5 ± 1.2	5.2 ± 0.8	4.8 ± 0.6
*Bacillus cereus Z2-7*	62 ± 2.6	+++	4.5 ± 1.43	5.4 ± 1.6	3.8 ± 0.8	3.2 ± 0.24

### Extraction of Antifungal Substances

**FIGURE 3:**
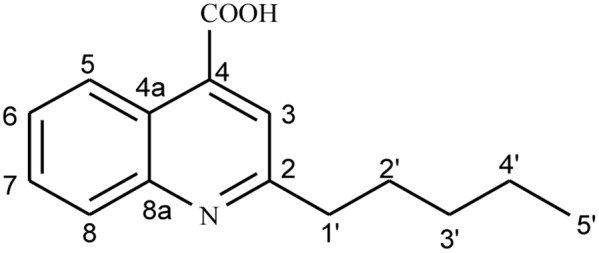
Structure of compound 1.

To further elucidate the antagonistic behavior of the bacterium against various fungi, supernatant from the fermented broth cultures were used to extract antifungal compounds by column chromatography. Thus, two compounds were isolated. Compound **1** was isolated as a cream crystalline powder from the EtOAc soluble fraction of bacterial supernatant of *L. sphaericus* ZA9 and gave positive test for alkaloids with DragendorffÏs reagent (Kim et al., 2009) (**Figure 3**). The molecular formula of **1** was assigned as C_15_H_17_NO_2_ on the basis on a quasi-molecular ion peak at *m/z* 266.1259 [M + Na] by ESIMS and spectral analyses.

**FIGURE 4:**
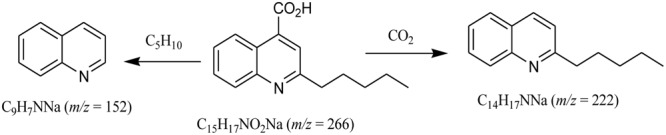
Key mass fragmentation pattern for compound 1.

The analysis of the ^1^H- and ^13^C-NMR data along with DEPT experiments revealed the presence of one CH_3_, four CH_2_, five aromatic CH, and five quaternary C-atoms in compound **1**. The ESI-MS indicated the presence of fragment-ion peaks at m/z 222 and 152 corresponding to the loss of CO_2_, and C_5_H_10_ groups, respectively, from the molecular ion of compound (**1**) (**Figure 4**).

The IR spectrum displayed absorption peaks at 1580 and 1420 cm^-1^, suggesting the presence of a benzene ring while a strong band at 3410 cm^-1^ supported the presence of OH group. The ^1^H-NMR spectrum of **1** exhibited signals for one Me group at δ_H_ 0.85 (*t*, 3H, *J* = 7.0 Hz)), four methylene groups at δ_H_ 1.32–134 (*m*, 4H), 1.57 (*m*, 2H), 2.61 (*t*, 2H, *J* = 7.0 Hz)), five sp^2^ methine H-atoms at δ_H_ 7.32 (*t*, 1H, *J* = 7.8 Hz), 7.38 (*d*, 1H, *J* = 8.4 Hz) 7.57 (*t*, 1H, *J* = 7.8 Hz), 8.33 (dd, 1H, *J* = 7.8, 0.6 Hz), and 6.18 (*s*, 1H) indicated the presence of basic quinoline skeleton. The ^13^C-NMR together with the HSQC experiment revealed 15 carbon resonances, which included one carbonyl signal (δ_C_ 178.8), four methylene signals (δ_C_ 34.4, 31.6, 29.6, and 28.5), five methine signals (δ_C_ 131.9, 125.9, 123.6, 117.2, and 108.8), one Me (δ_C_ 14.3), and four quaternary carbons (δ_C_ 165.1, 153.2, 139.7, and 124.9), which further supported the basic quinoline skeleton (Yin et al., 2010).

**FIGURE 5:**
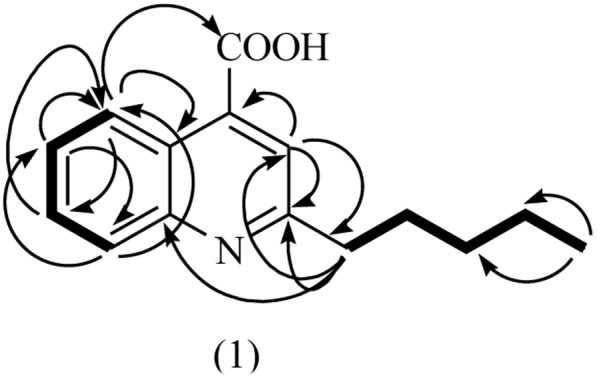
HMBC (H–C) and COESY (H–C) correlation for compound 1.

The HMBC correlation between H-atom (H-5) at δ_H_ 8.33 and C-atoms at δ_C_ 178.8 (C-9), 139.7 (C-4a), and 131.9 (C-7); H-6 at δ_H_ 8.33 and C-atoms at δ_C_ 125.9 (C-5) and 117.2 (C-8); H-7 at δ_H_ 7.57 and C-atom at δ_C_ 125.9 (C-5); H-8 at δ_H_ 7.38 and C-atoms at 125.9 (C-5) and 123.6 (C-6) showed the ortho-, meta-, and para-substitution in benzene ring. The cross-peak correlations of compound **1** in HMBCs between H-1′ at δ_H_ 2.61 and C-atoms at δ_C_ 108.8 (C-3), 165.1 (C-2′) and 153.2 (C-8a) and H-3 at δ_H_ 6.18 and C-atoms at δ_C_ 165.1 (C-2), 34.4 C(2′) and 124.9 (4) indicated the presence of long chain at C-2. A downfield signal at δ_H_ 11.98 (s, ^1^H) in ^1^HNMR and its direct correlation with C = O C-atom [H–C (3′)] in HSQC was assigned to carboxylic H-atom, while the HMBC correlation of H-5 with C = O (carboxylic acid) further supported the position of carboxylic group at C-4 (Kowsari and Mallakmohammadi, 2011). Assignment of ^13^C-NMR chemical shifts of compound **1** was completed with the help of HMQC, HMQC and DEPT experiments, which further supported the assigned substitutions at the 4-quinolinecarboxylic acid skeleton (**Figure 5**).

The proposed structure of **1** was further confirmed by comparison of the ^1^H- and ^13^C-NMR spectra obtained with those of a known 4-quinolinecarboxylic acid (Yin et al., 2010) with a difference in the substitution of alkyl group at C-2 position. Thus, all of the above evidences led to the elucidation of structure as 2-Penthyl-4-quinolinecarboxylic acid for compound **1**.

**FIGURE 6:**
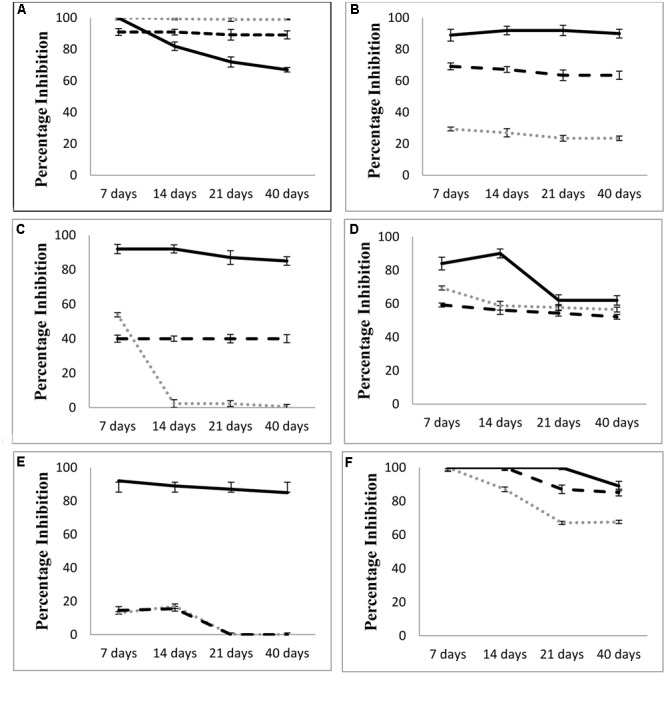
Comparison of antagonistic activity of Compound 1; 2 pentyl 4 quinolone carboxylic acid with *L. sphaericus* isolate and fungicide Benlate at different time points against **(A)** *Alternaria alternata*, **(B)** *Curvularia lunata*, **(C)** *Aspergillus* sp., **(D)** *Sclerotinia* sp., **(E)** *Bipolaris spicifera*, **(F)** *Trichophyton* sp.

Compound **2** was named as 1-methyl cyclohexene. Both compounds were evaluated for their antagonistic activity against phytopathogenic fungi as mentioned in **Figure 6**. Compound 1; 2-Penthyl-4-quinolinecarboxylic acid was found to be highly antagonistic against *A. alternata, C. lunata, Sclerotinia* sp., and *Trichophyton* sp. as compared to the whole bacterium (**Figure 6**) in a time course study. Compound 1 showed higher mycelial inhibition of *C. lunata*; *Aspergillus* sp., and *Trichophyton* sp. and lower mycelial inhibition of *A. alternata* as compared to the *L. sphaericus* ZA9 at different time points. However, the compound 1 had similar mycelial growth inhibition of *Sclerotinia* sp. and *B. spicifera* to that exhibited by the *L. sphaericus* ZA9 at all-time points. Fungicide Benlate remained highly antagonistic at varying levels against most of the fungi tested at different time points. Benlate showed greater inhibition of *C. lunata, Sclerotinia* sp., *Aspergillus* sp., *B. spicifera*, and *Trichophyton* sp. as compared to compound 1 and *L. sphaericus* ZA9 at all-time points. However, in case of *A. alternata*, compound 1 and *L. sphaericus* ZA9 exhibited higher mycelial growth inhibition as compared to Benlate which showed decline in mycelial growth inhibition with the passage of time.

Compound 2 didn’t show any antagonistic activity against any of the fungi tested.

## Discussion

Under natural environmental conditions plant growth and development is highly dependent on presence of beneficial microorganisms and absence of harmful ones in the surrounding (Glick, 1995). The beneficial microorganisms not only improve plant growth directly by nitrogen fixation, phytohormones production and P-solubilization but also by alleviating biotic and abiotic stresses. The present study evaluates the potentials of *L. sphaericus* ZA9 for plant growth promotion and biocontrol of phytopathogenic fungi.

Our results indicated that *L. sphaericus* ZA9 produces high levels of IAA and can solubilize insoluble minerals such as silicates, phosphates, and potash into soluble form probably by production of organic acids (**Table 1A**). This might be the reason for increased seed germination and seedling vigor of tomato and cucumber seeds pretreated with *L. sphaericus* ZA9 (Glick, 1995; Lugtenberg et al., 2002). Several mechanisms have been suggested for such observations involving other PGPB strains. These mechanisms include direct growth promotion by IAA production and increasing bioavailability of P viz. organic acid production and indirect growth promotion by eradicating phytopathogens (Berthelin and Belgy, 1979; Malinovskaya et al., 1990; Hiebert and Bennett, 1992; Barker et al., 1998).

Besides enhancing seed germination the bacterium *L. sphaericus* ZA9 significantly enhanced cucumber and tomato shoot length. However, at 21 dpp the root length of both tomato and cucumber seedlings pretreated with the bacterium *L. sphaericus* ZA9 was significantly less as compared to that of the uninoculated control and reference strain which could be due the fact that the bacterium is producing high quantities of IAA which results in decrease in primary root length. Exogenous IAA supply has been previously reported to regulate plant growth and development (Dobbelaere et al., 1999). Low IAA levels have been known to stimulate primary root elongation, whereas high IAA levels enhance the formation of lateral roots, increase root hair formation but decrease primary root length (Dobbelaere et al., 1999; Patten and Glick, 2002; Perrig et al., 2007; Spaepen et al., 2007; Remans et al., 2008).

Plant growth promoting biocontrol bacteria (PGPB) help the plant to combat phytopathogenic fungi by a myriad of mechanisms (Glick, 1995). We have reported here the bioantagonistic activity of *L. sphaericus* ZA9 against six phytopathogenic fungi in comparison to the reference strain *B. cereus* Z2-7 which is a known biocontrol strain (Naureen et al., 2005, 2009, 2015). The bacterial isolates exhibited moderate to high antagonistic activity against the tested fungi at various time points (**Table 2**). This could be due to higher amount of siderophores produced by test bacterium as compared to the reference strain which bind iron with a high affinity thus depriving the pathogen of iron (Naureen et al., 2005). Besides that the test bacterium produced high quantities of hydrolytic enzymes as compared to the reference strain. The hydrolytic enzymes damage the membrance and inhibit the spore formation (Beneduzi et al., 2012).

Plant growth promoting biocontrol may produce several secretory antifungal metabolites depending upon stage of bacterial growth, fermentation medium used; fungal secondary metabolites; nature of fungus; and mechanism of antagonism (Fernando et al., 2006; Wang et al., 2011; Naureen et al., 2015). The CFCS of both *L. sphaericus* ZA9 and *B. cereus* Z2-7 showed varied response against various fungi. This could be due to the reason that each bacterium produces different quantities of secondary metabolites and hydrolytic enzymes (**Table 3**).

The CFCS of *L. sphaericus* ZA9 exhibited strong antagonistic activity against *A. alternata, Sclerotinia* sp., *C. lunata*, and *Trichophyton* sp. as compared to *B. cereus* Z2-7 and it’s CFCS (**Table 2**). This confirms that the bacterium is secreting highly antagonistic extracellular antifungal metabolites in the medium besides siderophores and hydrolytic enzymes (Naureen et al., 2015). This was further confirmed by extraction of extracellular metabolites from the culture supernatant of the bacterium resulting in isolation of two main compounds. The ^1^H- and ^13^C-NMR spectra of compound 1 were compared with those of a known 4-quinolinecarboxylic acid (Yin et al., 2010) with a difference in the substitution of alkyl group at C–2 position. Thus, all the above evidences led to the elucidation of structure as 2-Penthyl-4-quinolinecarboxylic acid for compound **1** while compound 2 was recognized as 1 methyl cyclohexene. This study is the first ever report of production of 2-Penthyl-4-quinolinecarboxylic acid and 1 methyl cyclohexene from any bacterial source up to best of our knowledge. Moreover Compound 1 has not been reported previously from any biological source.

Both compounds were further evaluated for their antifungal activity against the aforesaid fungi. Compound 2; 1 methyl cyclohexene didn’t show any antifungal activity while Compound 1; 2-Penthyl-4-quinolinecarboxylic acid exhibited strong antagonistic activity against most of the fungi tested as compared to the test bacterium and its antagonistic activity is comparable to that of the fungicide Benlate (**Figure 6**). However, in case of *A. alternata*; 2-Penthyl-4-quinolinecarboxylic acid had slightly lower antagonistic activity as compared to whole bacterial strain which may be attributed to the myriad of mechanisms used by PGPB in combating phytopathogenic fungi while isolated antifungal metabolites may control fungi via a single possible mechanism (Naureen et al., 2015). This suggests that *L. sphaericus* ZA9 as well as the compound 1 isolated from its fermentation cultures both hold significant potential to be further elucidated as potent PGPB and biopesticide. Besides this the bacterium may also be investigated further in field conditions to confirm its suitability for development of biofertilizers.

## Conclusion

Our studies have indicated that *L. sphaericus* ZA9 has both plant growth promotion and biocontrol potential. The bacterium can improve plant growth by phytohormone and siderophore production; mineral solubilization, hydrolytic enzymes and antifungal metabolites. Moreover, the bacterium holds promise in production and development of natural fungicide to combat phytopathogens. Further studies will be carried out to evaluate the performance of the bacterium; its culture supernatant and the purified compound 1 in green house and field conditions.

## Author Contributions

Main theme, organization of work, isolation, characterization and identification of bacteria were done by ZN. Antagonistic assays were conducted by SAH under supervision of ZN. Isolation of antifungal metabolites and TLC was done by SAH under supervision of ZN and NR. Identification of antifungal metabolites were done ZN, HH, GA, AH, and JH. Si, P, K quantification was done by ZN, SF, and SG. Mass Spectroscopic analysis for antifungal metabolite was done by ZN and FM. IAA quantification by ELISA was done by ZN and AK. NMR facilities were provided by AH.

## Conflict of Interest Statement

The authors declare that the research was conducted in the absence of any commercial or financial relationships that could be construed as a potential conflict of interest.
